# DRIVE v3: Command Line Application for Identity‐by‐Descent Haplotype Clustering in Large Biobank Scale Data

**DOI:** 10.1002/gepi.70048

**Published:** 2026-06-26

**Authors:** James T. Baker, Hung‐Hsin Chen, Grahame F. Evans, Alyssa C. Scartozzi, Ryan J. Bohlender, Chad D. Huff, Quinn S. Wells, David C. Samuels, Jennifer E. Below

**Affiliations:** ^1^ Division of Genetic Medicine Vanderbilt University Medical Center Nashville Tennessee USA; ^2^ Institute of Biomedical Sciences Academia Sinica Taipei Taiwan; ^3^ University of Texas MD Anderson Cancer Center Houston Texas USA; ^4^ Department of Medicine Vanderbilt University Medical Center Nashville Tennessee USA; ^5^ Vanderbilt Translational and Clinical Cardiovascular Research Center Vanderbilt University School of Medicine Nashville Tennessee USA; ^6^ Department of Molecular Physiology & Biophysics Vanderbilt University School of Medicine Nashville Tennessee USA

**Keywords:** biobanks, identity‐by‐descent, population genetics, software

## Abstract

There is a need for genetic analytical methods that integrate multi‐individual identity‐by‐descent (IBD) tools with phenotypic enrichment testing to discover novel shared haplotypes contributing to disease traits. Existing tools are designed to identify IBD sharing and leave interpretation and phenotype association tests to further analyses. Here we present Distant Relatedness for Identification and Variant Evaluation (DRIVE) v3, a python command‐line interface tool that identifies networks of participants who share an identical haplotype at a given genomic location. Given phenotypic data, DRIVE additionally estimates significant enrichment of dichotomous traits within networks. DRIVE is designed for efficient use across large‐scale genetic data resources, featuring a versatile application programming interface and a backend structure designed for flexible integration into existing analytical pipelines. In this work, we describe the implementation of DRIVE v3 and illustrate two applications of the tool to an autosomal dominant condition and to an autosomal recessive condition, cardiomyopathy and cystic fibrosis, respectively. These applications highlight the substantial performance improvements between v1 and v3 and demonstrate practically how the newer features of DRIVE such as the enrichment test can be used in the interpretation of the identified networks.

## 1 Introduction

Identity‐by‐descent (IBD) segments are regions of the genome shared without recombination by individuals due to inheritance from a common ancestor. IBD is leveraged in numerous genetic analyses, including pedigree reconstruction (Evans et al. 2025; Staples et al. 2014; Staples et al. 2016), pairwise relatedness estimates (Huff et al. 2011), genotype imputation (Abney and ElSherbiny 2019), quantifying mutation and gene conversion rates (Browning and Browning 2024; Palamara et al. 2015), and IBD mapping which maps disease genes to phenotypes based on enrichment of pairwise segmental sharing among cases (Browning and Thompson 2012; Mahmoudiandehkordi et al. 2024).

Most methods that employ IBD focus on pairwise segment sharing, ignoring the additional information that can be gained by considering multi‐individual IBD. Examples of such programs include iLASH (Shemirani et al. 2021), hap‐IBD (Zhou et al. 2020), GERMLINE (Gusev et al. 2009), and FASTSMC (Nait Saada et al. 2020), which detect pairwise shared IBD segments, and ERSA (Huff et al. 2011), which estimates distant relatedness based on the abundance and length of pairwise IBD segments. A few tools have been developed which aggregate pairwise IBD information, including DASH (Gusev et al. 2011), ibd‐cluster (Browning and Browning 2024), and FoundHaplo (Robertson et al. 2025). The goal of DASH is to discover rare variant associations via graph theory by aggregating pairwise IBD segments into multi‐sample haplotype clusters. DASH identifies these multi‐individual IBD clusters but lacks any integrated functionality for phenotype association or enrichment testing. Instead, the recommended approach for DASH is to use PLINK (Chang et al. 2015) to compare the rate of phenotype cases within a cluster to the rate outside the cluster across the genome (Gusev et al. 2011), a strategy with a high burden of multiple testing. ibd‐cluster also identifies multi‐individual IBD clusters with the stated purpose of making IBD detection and storage more efficient compared to pairwise IBD. Due to the explicit focus on efficiency, ibd‐cluster lacks integrated statistical methods for identifying segments significantly associated with phenotypes. All of these tools identify haplotypes genome‐wide. In contrast, FoundHaplo tests for sharing of a previously identified haplotype of interest (identified using some external method) forming a multi‐individual IBD cluster. Collectively, these tools identify IBD sharing, but leave interpretation and phenotype association tests to further analyses. The restrictions of these programs highlight the need for tools that aggregate multi‐individual IBD and integrate phenotype enrichment statistical tests to prioritize haplotypes for further investigation. These tools need the extensibility to easily incorporate a broad range of additional modules for further investigation.

**Figure 1:**
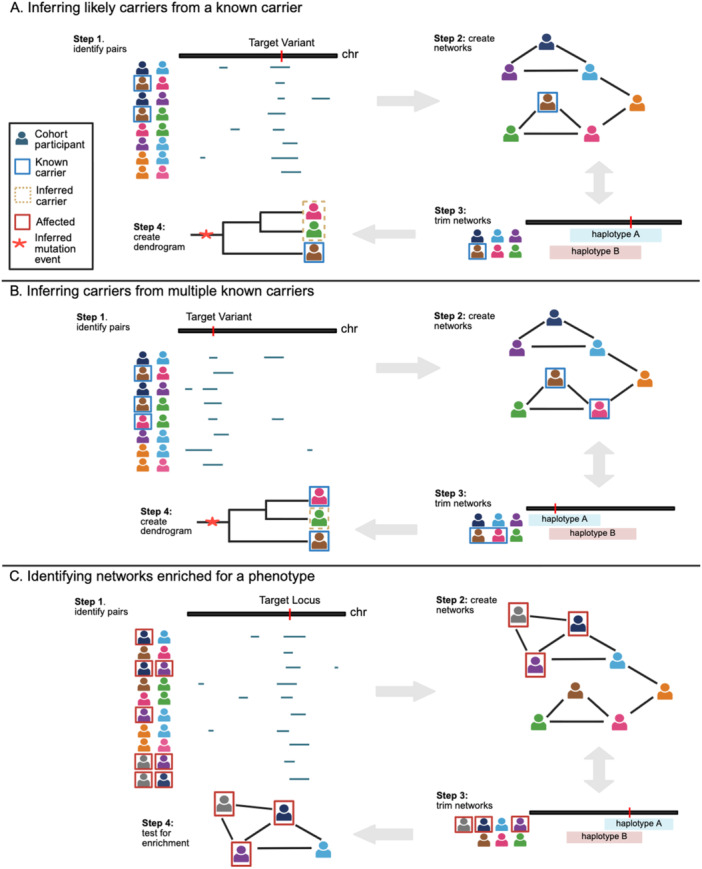
Conceptual illustration of use cases of DRIVE. *Panels A* and *B* illustrate how DRIVE can be used to infer likely carriers from known carriers. In both panels, the steps can be interpreted as follows*. Step 1*. DRIVE filters the shared pairwise IBD segments from the input cohort to those overlapping the target locus. *Step 2*. DRIVE identifies networks of individuals who share these IBD segments. *Step 3*. DRIVE trims sparse networks and repeats the network identification. *Step 4*. The inverse of the IBD segment length is used to represent genetic distance to form the dendrogram. Mutation events can be inferred by integrating the dendrogram with sequencing information. *Panel C* illustrates how DRIVE can be used to identify networks of individuals which are enriched for a phenotype of interest. *Steps 1–3* are the same as in Panels A and B except information about case/control status is provided instead of carrier status. In *Step 4*, DRIVE tests for phenotypic enrichment using binomial statistics comparing the network to the total cohort.

To address this need, we introduce DRIVE (Distant Relatedness for Identification and Variant Evaluation) v3, an open source IBD‐based tool that can be used both to infer additional carriers of a rare variant given a known underlying haplotype (Figure 1A,B) as well as identify clusters of individuals who share a genomic segment in a given locus and are enriched for a disease of interest, suggesting the presence of a previously unknown causal variant on the shared segment (Figure 1C). DRIVE is distinct from other tools and has expanded utility. It efficiently leverages IBD to identify networks of individuals with shared haplotypes at a specified locus and includes a process for trimming sparse networks. An early version, DRIVE v1, was used in Lancaster et al to identify additional likely carriers of a rare variant in *KCNE1*, D76N, causing a heart rhythm disorder, long QT within sparse genotyping array data (Lancaster et al. 2024). Starting in v3, DRIVE includes a new set of features aiming to improve performance, ease of use, and integration into existing pipelines. These features include the ability to estimate enrichment of dichotomous traits within networks, a 27x improvement in speed compared to v1, the ability to construct dendrograms within networks which cluster individuals based on the length of the genomic segment they share at a locus, and a new flexible backend architecture that allows users to connect their own custom code to DRIVE to adjust the program's function. In this work, we describe the implementation of DRIVE, its inputs and outputs, the main clustering algorithm, the DRIVE enrichment test, dendrogram generation, and the architecture of DRIVE that enables it to be customizable and versatile. Additionally, we illustrate the application of DRIVE in a biobank for two established genetic conditions: an autosomal recessive condition, cystic fibrosis (CF) and a commonly autosomal dominant condition, cardiomyopathy. These two applications demonstrate how to interpret the networks identified by DRIVE and showcase the tool's ability to identify haplotypes containing causal variants.

## 2 Materials and Methods

### 2.1 DRIVE Haplotype Clustering Algorithm

DRIVE first filters the provided IBD inputs using both a basepair position window and a centimorgan threshold for the segment length. The base position restricts the analysis to only those segments overlapping the provided target locus start and end position. Segments are also filtered to only those longer than a user‐provided minimum segment length threshold (default 3 cM). IBD detection software, such as hap‐IBD or iLASH, determines segment length by mapping the endpoints of the pairwise IBD segment to centimorgans using a recombination map. A graph of the data is then constructed where each participant's phased haplotype is a vertex, and each edge represents a shared IBD segment. When a person shares a segment with themselves at the locus, they are represented by two vertices labeled ID.1 and ID.2 in one network, where ID is the participant's study identifier. In circumstances where individuals may share multiple IBD segments within a target locus, each shared segment is represented as an edge within the graph. This situation is unlikely to occur for small loci such as a gene but could occur as a result of recombination or if a single IBD segment spanning the target locus was incorrectly fragmented and detected as several smaller segments. Once a graph is constructed, DRIVE uses an iterative process to traverse this graph and identify networks. This graph is first traversed using a community walktrap algorithm, where the segment length is used as a probability weight for each edge.

This algorithm calculates a transition probability matrix for the graph, where each value represents the probability of traversing from one vertex to another. Vertices are aggregated into “communities” using a hierarchical agglomerative clustering algorithm. The community walktrap algorithm allows for the network identification to be deterministic between runs. This algorithm and the mathematical proofs can be found in Sections 2 and 4 of Pons & Latapy (Pons and Latapy 2006). To reduce identification of spurious large networks connected by very small segments, the size and connectedness of each network are calculated, where connectedness is defined as the number of edges in the graph divided by the number of total possible edges. For large (default size ≥ 30) and sparsely connected networks (default < 50% connected), DRIVE will iteratively execute a re‐clustering process for each network that mimics the original traversal using a new graph consisting of only the haplotypes within the network. If the original network is not broken up by the additional walktrap, DRIVE then performs a hub detection algorithm that calculates how many edges each node has and generates a penalization score for each node by summing the inverse IBD segment length for every pairwise segment involving that node. Nodes are then classified as hubs if they are connected to a substantial proportion of other nodes in the network (default ≥ 20% of network size) and if the penalization score falls in the tail of the distribution for the network (default ≥ top 1% of scores). These identified hub nodes are all removed from the graph and another community walktrap is performed. This re‐clustering process repeats until all networks meet the provided size and sparsity requirements, or the number of iterations exceeds the provided limit (default 5, Figure S1). This process is illustrated in the “Hub Detection algorithm” section within the supplemental data (Figures S2–S5).

### 2.2 Enrichment Test

DRIVE tests for enrichment of phenotypes within networks using a binomial test (Figure S1) for which the null hypothesis is that the frequency of the phenotype in the network is no different than the frequency in the overall cohort. Only networks containing at least two phenotype cases are tested, with one case treated as a proband for each network and dropped from the following statistical calculation. The phenotype frequency within each network is then compared to the frequency within the entire cohort using the binomtest function in the python scipy.stats module to generate an enrichment p‐value. A multiple test correction can be calculated based on the number of networks that were tested (Equation 1).

(1) $$\mathrm{adjusted}\;p\;\mathrm{value}\;\mathrm{threshold}=\frac{0.05}{\#\;\mathrm{of}\;\mathrm{networks}\;\mathrm{containing}\geq 1\mathrm{case}}$$

If sequencing data is available, identification of a rare pathogenic or likely pathogenic variant within the haplotype can provide additional support for causality.

### 2.3 Dendrogram Creation

DRIVE visually illustrates how individuals within each network cluster based on the length of the pairwise shared IBD segments by generating a dendrogram for a network through agglomerative hierarchical clustering using Ward's method (Ward 1963). The branch length between each pair of individuals is calculated from the inverse length of the shared IBD segment (in cM) overlapping the targeted locus. Typically, longer shared IBD segments indicate closer relatedness between the two individuals, who are placed closer together on the dendrogram. More information about optional inputs to generate the dendrogram is described on the page “DRIVE Inputs” within the online documentation.

### 2.4 Implementation

DRIVE (3.1.0) is a command‐line tool implemented in Python (≥ 3.10). DRIVE incorporates common data science libraries such as Pandas (≥ 1.5.3), Numpy (≥ 1.24.2), iGraph (≥ 0.10.4), DuckDB (Raasveldt and Mühleisen 2019) (> = 1.4.2), and Scipy (≥ 1.10.1) all of which implement their core functionality in low‐level languages such as C, C + +, and Fortran for performance. Matplotlib (≥ 3.9.4) is used for generating plots. DRIVE has been packaged on the Python Package Index as “drive‐ibd” and is also available in a containerized image on DockerHub to ensure usability on both unix‐like and cloud environments. DRIVE has been tested on the Ubuntu 24.02 LTS operating system and MacOS Ventura 13.3 but is designed with a platform independent framework to ensure widespread compatibility. Example commands for running provided test data can be found in the “Testing DRIVE” section of the online documentation.

### 2.5 Inputs Options

DRIVE is compatible with output files from the following IBD detection software: hap‐IBD, iLASH, GERMLINE, and RaPID (Naseri et al. 2019). These files can be either uncompressed or compressed (gzip or zstandard compression). DRIVE also requires that a target region or position in the genome be provided as a character string. This target locus can be a variant, gene, or a region of interest from either genome‐wide association studies (Klein et al. 2005) or IBD mapping. DRIVE uses this locus to filter to pairwise IBD segments that either overlap this user‐defined locus or that contain the entire locus (this behavior can be customized with the *“‐‐segment‐overlap”* flag). Memory usage and runtime are correlated with target region size for target regions larger than the minimum shared segment length. A larger target locus increases the number of potential pairwise segments overlapping the region which correlates to more time and memory needed to construct a larger graph with more connections. Due to this consideration, we recommend that the target locus be no larger than a few megabases. Details on the influence of other parameters on runtime and memory are described in the “Influences on runtime and memory behavior” section with the supplemental.

The user may provide a tab‐separated phenotype file containing one column with participant IDs and one or more columns for binary phenotypes. If this file is provided, DRIVE will only include individuals in the phenotype file within the analysis. DRIVE will use the phenotype status to perform an enrichment test for each phenotype within every identified network using a binomial test. This enrichment test is described further in Section 2.2. The phenotype input file format is flexible and may be as small as a single targeted phenotype or large, such as for a Phenotype‐Wide Association Study (PheWAS) (Denny et al. 2010). When no phenotypic data is provided, DRIVE will only perform the network identification.

### 2.6 Output Files

The output of DRIVE is a tab‐separated text file where each row describes network properties, including size, connectedness, participant IDs, and labels of phased haplotypes, formatted as “*participant_id.1 or participant_id.2.”* At most, each participant will belong to two networks within this file (one for each phased haplotype). If the user provides a phenotype file as input, then the output file will also contain five additional columns for each provided phenotype. These columns list the ids for individuals in the network who were classified as cases, those ids classified as exclusions based on the phenotype file, the corresponding counts of case and exclusion ids in the network, and the calculated p‐value for the phenotype. DRIVE also outputs a log file in the output directory of the analysis to preserve analysis details and runtime information.

### 2.7 Parameters and Flags to Customize Behavior

The community walktrap algorithm of DRIVE can be tuned using several parameters that affect either the community detection, the iterative re‐clustering procedure, the thresholds used by the hub detection algorithm, or the IBD segments filtering. Exact flags that affect these behaviors are described in Supporting Information S1: Table S1. By default, DRIVE only records basic runtime information to an output log file. The user can provide runtime flags to customize the verbosity of DRIVE so additional information is passed to standard output as well as a log file. Further information regarding permissible flags for DRIVE is described in the section of the online documentation titled “DRIVE Inputs.”

### 2.8 Plugin Architecture and Extensibility

To allow flexibility for other analytical pipelines, all steps after the clustering algorithm in DRIVE were implemented using the “plugin” (Mayer et al. 2003) object‐oriented design pattern. The plugin design pattern allows smaller, custom programs to be tightly integrated into larger programs to add new and custom functionality. DRIVE incorporates this pattern by providing all identified networks through an API endpoint. Users can optionally design their own “plugins” that interface with this API to add custom functionality or to change existing functionality. DRIVE comes with two default plugins defined in a JSON file, which are used for the enrichment test and to write the main output file (Figure S1). The user can create a new JSON file with custom plugins, which will be used during runtime. Additional information regarding the data provided through the API and the required format of any custom plugins is described in the section of the online documentation titled “Extending DRIVE.”

### 2.9 Biobank Data Used in the Examples

The Alliance for Genomic Discovery (AGD) is a partnership between Vanderbilt University Medical Center (VUMC), Illumina, and Nashville Biosciences which carried out whole genome sequencing on 250,000 individuals with deidentified electronic medical records data in the VUMC biobank BioVU. Sequenced variants were called using the DRAGEN Germline Variant Calling pipeline (v3.7.8) using the hg38 reference panel. The variant set was then recalibrated using the DRAGEN Machine Learning Recalibration pipeline (v4.2.4), and results were aggregated using the DRAGEN Iterative gVCF Genotyper pipeline (v.4.3.7). The Illumina Connected Analytics cloud platform was used for all the above variant calling and QC.

### 2.10 Detecting Pairwise IBD Segments in the AGD

To generate the pairwise IBD segments as inputs for DRIVE, we filtered variants in the AGD WGS to those that were bi‐allelic, had a cohort minor allele frequency threshold to common variants (cohort frequency ≥ 1%), a variant missingness rate ≤ 5%, a sample missing rate ≤ 10%, and those variants on Illumina's MEGA^EX^ genotyping array. These filtering and extraction steps were performed using BCFtools (Danecek et al. 2021) and PLINK2 (Chang et al. 2015). These variants were then phased using SHAPEIT5 (Hofmeister et al. 2023) with default parameters. Pairwise shared segments were then detected in the phased data using hap‐IBD with default parameters. This data was then provided as input for the network analysis using DRIVE (3.1.0).

### 2.11 Phenotyping of Cystic Fibrosis and Cardiomyopathy

To phenotype for cystic fibrosis (CF) and cardiomyopathy, we first mapped the ICD9 and ICD10 codes from VUMC's EHR to phecode 1.2 using the python PheTK package (Tran et al. 2024). All individuals with two or more occurrences on unique dates of the CF phecode 499 or the cardiomyopathy phecode 425 were classified as cases for each analysis. Individuals with only one occurrence of the respective code were classified as exclusions. These individuals were clustered into networks but were excluded from statistical analysis All other individuals were classified as controls for the analyses.

### 2.12 Benchmarking DRIVE v3 Against DRIVE v1

To compare DRIVE v1 to DRIVE v3, we installed both versions of DRIVE into Python virtual environments using pip and the venv module. We ran DRIVE v3 using Python 3.12. We ran DRIVE v1 using python 3.9, which is the most recent version that is supported. DRIVE v1 relies on the “distutils” package and Pandas v1.1.5 which were either removed from or are not supported by Python version > 3.9. Both tests were run on Ubuntu 24.04.2 LTS and were profiled using the unix tool “time.” Runtime was reported by both “elapsed wall clock time” and “User time” (CPU usage). Memory was reported as the “Maximum resident set size.” Both versions of DRIVE were then run on the *CFTR* gene locus using the AGD pairwise shared segment data to identify networks of individuals who shared haplotypes overlapping *CFTR*. Since DRIVE v1 does not support any enrichment testing, only the network identification and network pruning steps of DRIVE v3 were performed for the comparison. Default parameters were used for both programs. The runtime performance of each program was measured using the Python cProfile module and visualized using SnakeViz.

Additionally, we identified quantified how many networks identified by v1 fully reproduced in v3 as an additional comparison metric. For each network identified by DRIVE v1, we generated a string by concatenating the sorted haplotype ids. Each string was stored in a set. We repeated this process for the networks identified by DRIVE v3. We took the intersection between these sets to see how many networks were identified in common. We restricted this comparison to networks of size ≥ 3 as DRIVE v1 was unable to identify networks of size 2.

## 3 Results

### 3.1 Comparison of DRIVE v3 to DRIVE v1

When both versions of DRIVE were used to identify networks containing the *CFTR* locus, we observed a ~ 35x performance increase using DRIVE v3 (Table 1). DRIVE v3 identified 58,082 networks (including networks of size 2) in 1 h and 3 min using 7.9 GB of memory, while DRIVE v1 identified 35,113 networks in 37 h and 14 min using 3GB of memory. DRIVE v1 is limited to identifying networks of a minimum size 3, while DRIVE v3 has capability of reporting networks of size 2 as well, explaining the substantial increase in number of networks identified. DRIVE v1 identified 35,113 networks of size ≥ 3. DRIVE v3 fully recapitulated these networks.

**Table 1:** Comparison of runtime and memory for DRIVE v1 and DRIVE v3 when used to identify networks of individuals who share an IBD segment overlapping the CFTR locus in the AGD250k dataset.

Profiling category	DRIVE v1	DRIVE v3
Runtime (Wall clock)	36 h 16 m	1 h 3 m
Runtime (CPU usage)	20 h 25 m	1 h 11 m
Memory (Gb)	3.1	7.9

### 3.2 Inheritance Pattern Effects on DRIVE

The inheritance pattern of a disease affects the interpretation of DRIVE results. The simplest case is autosomal dominant diseases, where a single copy of the causal allele can cause disease. Incomplete penetrance (presence of the causal variant without the disease manifesting) of dominant effects will reduce power. Autosomal recessive diseases, which require both copies of a gene to be disrupted to manifest risk, are more complex to analyze with DRIVE since the presence of two, sometimes distinct (e.g., compound heterozygosity), deleterious variants must be considered. Although not illustrated in this work, DRIVE could additionally be run for X‐linked traits. Since DRIVE only considers one of the two haplotypes for a participant at a time during the graph construction and traversal, the algorithm behind the network identification and refinement steps would remain the same.

### 3.3 Application of DRIVE for an Autosomal Dominant Disease

To demonstrate how DRIVE could be used for autosomal dominant conditions, we ran DRIVE in the AGD cohort targeting the *TTN* locus on chromosome 2. *TTN* encodes the protein titin, which provides stiffness to the striated muscle sarcomere and modulates active contractile force (Herman et al. 2012). DRIVE identified 56,040 networks overlapping the *TTN* locus, ranging from size 2–702 participants with a median size of 3. Truncating mutations in *TTN* are among the most common causes of dilated cardiomyopathy (DCM), accounting for up to ~25% of cases (Herman et al. 2012; Kellermayer et al. 2019; Roncarati et al. 2013). Our analysis uses the more heterogeneous phecode for cardiomyopathy, which includes DCM and other forms of cardiomyopathy such as hypertrophic cardiomyopathy, etc. This definition of cardiomyopathy classified 7895 participants as cases, 2618 as exclusions, and 237,957 participants as controls giving us a cohort frequency of 3.2%. There were 9589 networks which contained ≥ 1 cardiomyopathy case and 2306 networks which contained ≥ 2 cases. The most enriched network from the analysis consisted of 36 participants where 10 individuals were classified as cardiomyopathy cases giving a network frequency of 31.2% and an enrichment p‐value of 6.95e‐7. Exploring the whole genome sequencing data for the 34 participants, including the 10 cases, within this network revealed all but two to be heterozygous for the known pathogenic splicing variant chr2:178559309:A:T, also denoted as *TTN* c.81898+2 T > A (Norton et al. 2013). Cardiomyopathy cases were spread throughout the dendrogram, not clustered, which can be interpreted as an incomplete penetrance of the cardiomyopathy phenotype for carriers of this known pathogenic variant, consistent with clinical experience (Gigli et al. 2025).

**Figure 2:**
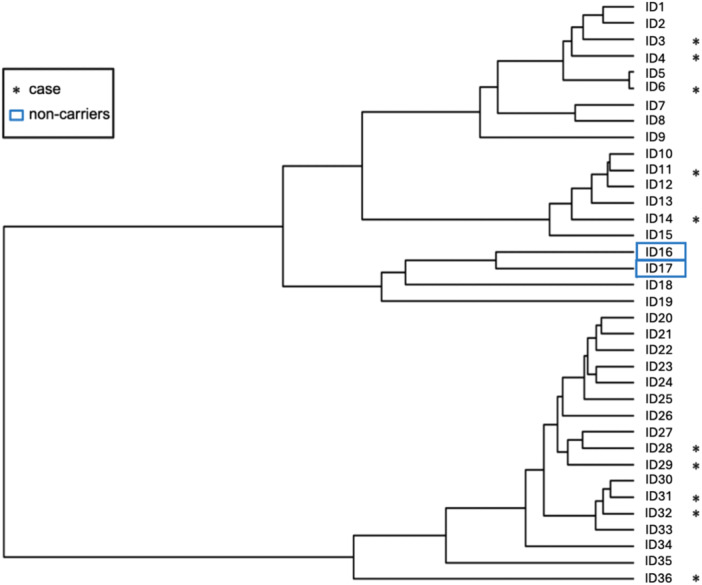
Dendrogram of local genetic distance calculated the inverse pairwise IBD segment length for enriched Cardiomyopathy network.

The two participants who did not carry this pathogenic variant were clustered in a single interior branch, not at the edge, of the dendrogram (Figure 2). While one explanation for this finding would be sequencing error, the GQ scores were 81 and 83, and the local allele depth values were 49 and 51 for these two participants, respectively. These higher scores suggest the calls are likely reliable. Other explanations include a back‐mutation to the reference allele or a double recombination event. This example only analyzed the most significant phenotype‐enriched network. In practice, a discovery analysis should be carried out separately for each network exhibiting phenotype enrichment, to a threshold determined by the user.

### 3.4 Application of DRIVE to Autosomal Recessive Diseases

To demonstrate how DRIVE can be used for autosomal recessive conditions, we ran DRIVE to identify networks of participants who overlapped the *CFTR* locus and tested for an enrichment of CF cases within each network. *CFTR* encodes a transmembrane protein that serves as an ion channel, notably for chloride ions. Variants in this gene often affect protein folding and cause dysregulation of Cl^−^ conductance (Trouve et al. 2024). The phenotype file used in this analysis did not contain 1605 individuals that were present in the DRIVE versions profiling, leading to the slightly smaller number of networks identified by this run of DRIVE. Our phenotyping approach using the CF phecode (499) classified 541 participants as cases, 231 as exclusions, and 247,698 as controls giving a cohort frequency of 0.2%. DRIVE identified 57,793 networks with a median size of 3 and ranging in size from 2 participants to 632. Of these 57,793 networks, 523 networks contained ≥ 1 CF cases, while 166 of the networks contained ≥ 2 CF cases. The most enriched network from the analysis consisted of 195 participants where 17 are cases, giving a network prevalence of 8.7% and enrichment p‐value of 1.68e‐20. From the AGD sequencing data, all 17 cases in this network were identified as carriers for the common and well‐characterized pathogenic variant p.Phe508del (ΔF508) (Kerem et al. 1989). This variant is by far the most commonly observed pathogenic CF variant (Sosnay et al. 2013) with a population frequency of 1.5% reported in gnomAD for non‐Finnish Europeans and is likely to be fairly old (> 50,000 years) (Kaplan et al. 1994; Morral et al. 1994). Haplotypes grow smaller over time as recombination events break down linkage disequilibrium between nearby variants, eventually becoming undetectable as they shrink below the length threshold of detection. All 195 individuals in this network were sequenced as carriers of the ΔF508 variant, indicating this variant was on the haplotype defining the network. Twelve of the 17 CF cases were homozygous for the ΔF508 variant. One of the homozygous ΔF508 carriers shared the IBD locus at this location with themselves and was thus present as two vertices in the network. Four of the five remaining cases were heterozygous for an additional pathogenic variant, with each having a different second pathogenic variant (chr7:117587778:G:T, chr7:117587811:C:T, chr7:117587806:G:A, chr7:117590440:G:A). The remaining case was not a carrier of any additional known pathogenic variant within the CFTR locus. Manual review of the EHR text for this individual indicated that they were never in fact diagnosed with CF but had received the relevant ICD code at least twice.

From this sequencing data alone, we were not able to determine if the additional heterozygote variant was in cis (on the same chromosome copy) or trans (on the second chromosome copy) to the ΔF508 variant. For an autosomal recessive disease such as CF, it is most common for the second pathogenic variant to be in trans with the first variant. To test if this is true, we examined the additional networks containing the four heterozygote cases to see if the haplotype for those respective networks contained the additional variant, as every individual can appear in at most two networks, or one for each copy of the chromosome.

### 3.5 Phenome‐Wide Analyses With DRIVE

The two examples above each examined IBD sharing at a known gene for a single phenotype. To illustrate the phenome‐wide analysis capability of DRIVE, we ran DRIVE targeting the *CFTR* locus with enrichment testing for the 1817 phenotypes defined in Phecode 1.2. In this example, we report the PheWAS results for the network of 195 individuals enriched for CF, described above. This network was additionally enriched for common CF comorbidities such as pseudomonal pneumonia, bacterial pneumonia, bronchopneumonia and lung abscess, and diseases of the pancreas (Table 2), illustrating how DRIVE can be used to identify the phenotypic spectrum of a disease among haplotype carriers.

**Table 2:** 10 most enriched phecodes when DRIVE was run phenomewide to identify networks overlapping the CFTR locus.

Phecode	Description	*p*‐value
499.0	Cystic fibrosis	1.71e–20
480.12	Pseudomonal pneumonia	3.12e–13
480.1	Bacterial pneumonia	6.9e–10
480.5	Bronchopneumonia and lung abscess	1.61e–8
480.13	MRSA pneumonia	6.33e–8
577.0	Diseases of the pancreas	9.57e–8
475.0	Chronic sinusitis	1.47e–5
496.2	Chronic bronchitis	3.10e–5
530.1	Esophagitis, GERD, related diseases	4.55e–5
530.11	GERD	4.83e–5

## 4 Discussion

DRIVE v3 is an open‐source Python tool utilizing IBD and graph theory in a biobank setting. Its strengths compared to competing software are (1) the ability to utilize multi‐IBD and identify networks defined by this multi‐IBD, (2) the ability to prioritize networks enriched for a phenotype(s) of interest, (3) the use of a plugin system that allows users to seamlessly integrate their own code to extend DRIVE to additional capabilities, (3) packaging through both PYPI and Docker, allowing for use on multiple CPU architectures and cloud environments, and (4) the ability to detect enrichment for dichotomous traits.

Recent developments in the Python language highlighted the need for substantial restructuring of DRIVE from v1 to v3. Due to the removal of the “distutils” package by the Python core team (Dower 2020) and the incompatibility of Pandas v1.1.5 with newer versions of Python, DRIVE v1 was unable to be used on any version of python newer than 3.9. This inflexibility meant DRIVE was unable to utilize many of the likely performance improvements brought by Python version 3.11+ and Pandas version 2.0 + . DRIVE v3 addresses these concerns by being compatible with newer version of python starting at 3.10. In addition, the lack of portability of DRIVE v1 made it difficult to use with many of the cloud‐based platforms such as All of Us, UK Biobank Research Analysis Platform, and Terra.bio. DRIVE v3 addresses this issue also by providing a containerized image ensuring portability and stability of the code.

DRIVE v3 demonstrated a 35x performance enhancement when compared to DRIVE v1 in our biobank of 250,000 people. DRIVE v1 read data in line by line and concatenated each line to a DataFrame causing CPU pressure through memory allocations, considering 85.3% of the total runtime was spent in this step (Figure S6A). A large difference was also observed between CPU time and wall clock time (20 h 25 m vs 36 h 16 m, respectively), indicating I/O pressure likely from the single‐threaded gzip decompression while reading in the data. The runtime of this step was reduced to 6.38% (Figure S6B) in DRIVE v3 by using the in‐memory database DuckDB. DuckDB is written in C + + and allows for multiple threads to be used to read and filter the IBD segment inputs. These performance improvements shifted the CPU bottleneck from the IBD segment filtering to the network identification and refinement steps, which consume 93.2% of the runtime (Figure S6B). These modules utilize many of iGraph's core C functions through a python API. This improved runtime of DRIVE enables users to run the tool in modern biobanks that have sequencing for hundreds of thousands of people.

Using DRIVE, we were able to identify networks of participants overlapping the *CFTR* and *TTN* loci that were enriched for CF and cardiomyopathy, respectively. These examples illustrate how the networks identified by DRIVE can be utilized to identify causal pathogenic variants underlying the network haplotype. Additionally, we illustrated the additional steps required to interpret DRIVE results for an autosomal recessive condition such as CF.

In addition to the two examples illustrated in this work, which demonstrate how to run DRIVE v3 by starting with a phenotype of interest (Figure 1C), DRIVE can also be used to identify enrichment of a variant of interest (Figure 1A,B). This function of DRIVE v1, which is retained in v3, was previously utilized to identify and investigate clinical consequences of carriers of a known *KCNE1* mutation in BioVU (Lancaster et al. 2024; Roden et al. 2008). Mutations in *KCNE1*, a gene which encodes the auxiliary subunit of the *I*_*Ks*_ conducting channel, have been linked to Long‐QT syndrome (Erlandsdotter et al. 2023). DRIVE v1 was used to identify 12,356 networks of participants who shared haplotypes containing the *KCNE1* locus. DRIVE identified 2 networks containing 23 BioVU participants where 22 of the 23 participants carried the known pathogenic *KCNE1* variant, p.Asp76Asn, confirmed by whole exome sequencing. This variant has been shown to delay cardiac repolarization and contribute to an increased risk of arrhythmias (Splawski et al. 1997).

When rare diseases are caused by the cumulative effect of many very recent mutations, within the last 1–2 generations, with different mutations in the same gene in different disease cases, relatively little multi‐IBD around the gene locus is expected but there is a much larger proportion of pairwise IBD at that gene (i.e., a large number of small IBD clusters). In this scenario, DRIVE will be unlikely to detect an enrichment of disease at this locus. In this situation, a method like IBD mapping that tests at the proportion of pairwise IBD by locus, aggregating evidence across distinct shared segments, may be better powered for locus effect discovery. Another limitation of DRIVE is its inability to detect enrichment from *de novo* mutations. *De novo* mutations would not be shared by any other individuals in the network and therefore there would be no phenotype enrichment within that network.

Additionally, the binomial enrichment test used in DRIVE is limited to dichotomous phenotypes. Future versions of DRIVE will be extended to support continuous traits through a non‐parametric test such as the Wilcoxon rank sum test.

DRIVE requires shared IBD segments identified by other software such as hap‐IBD, iLASH, GERMLINE, or RaPID. These programs have differences in how they identify shared IBD segments, including their tolerance for mismatched variants in defining a shared IBD segment. Different input data can alter the results of the DRIVE analysis. In comparing these tools for identifying IBD segments, we find they differ in the specific endpoints identified for the shared IBD segments but generally identify the same set of segments. Specific comparisons of these methods can be found in their associated manuscripts (Gusev et al. 2009; Naseri et al. 2019; Shemirani et al. 2021; Zhou et al. 2020). Since the minimum length used for detecting shared segments is much larger than the size of a gene, changes in the endpoints of a shared IBD segment do not often remove a target gene locus from the shared IBD segment leading to relatively minor changes in the networks of individuals identified by DRIVE.

DRIVE v3 extends the utility of IBD‐based discovery by integrating the additional information of multi‐individual IBD with enrichment testing, enabling users to prioritize haplotypes. This is a particularly useful test when causal variants are not directly measured as is often the case in array genotyping data (this is particularly relevant for array genotyped biobanks such as Our Future Health (Cook et al. 2025)), exome sequencing data, and for a range of variant types not well captured by common short read sequencing approaches (e.g., repeat expansions, structural variants). As the number of biobanks with sequencing data continues to increase, users will then be able to investigate these prioritized haplotypes to identify potentially causal pathogenic variants, including across biobanks. In addition, DRIVE's extensibility allows users to utilize the identified haplotypes in any analytical pipeline without having to change the DRIVE source code. Finally, the availability of DRIVE on both the PYPI registry and DockerHub allows the tool to be run in a wide variety of OS environments (MacOS, Linux, Windows) or in the modern cloud environments such as UKBB's Research Analysis Platform or Terra.bio.

## Conflicts of Interest

The authors declare no conflicts of interest.

## Supporting information

Supporting File

## Data Availability

Data sharing not applicable to this article as no datasets were generated or analyzed during the current study. DRIVE (v3) is available for download through the Python Package Index, PyPI, as “drive‐ibd” and as an image on DockerHub (https://hub.docker.com/r/jtb114/drive). The data used for testing DRIVE is bundled with both installations and commands for running the test are on GitHub. The source code for DRIVE and all data used for running tests are also available directly from GitHub (https://github.com/belowlab/drive). Extensive detail on DRIVE, its inputs and outputs, and the different installation methods can be found at https://drive-ibd.readthedocs.io/en/latest/. The genetic and clinical data used in this study are available through the Vanderbilt biobank (BioVU), comprised of DNA samples and de‐identified electronic medical records from consented participants. Access to the individual‐level data in BioVU requires IRB approval and is subject to Vanderbilt University Medical Center policies.
