# Supplementary material for: DRIVE v3: Command Line Application for Identity‐by‐Descent Haplotype Clustering in Large Biobank Scale Data
Source: Genet Epidemiol. 2026 Jun 26;50(5):e70048. doi: 10.1002/gepi.70048 (PMC13309748; doi:10.1002/gepi.70048)
Supplement: Supplementary file 1 — Supporting File [file GEPI-50-0-s001.docx]

**Supplemental Data:**

**Figure S1**. Flowchart of the DRIVE algorithm. Optional inputs are indicated by “*”


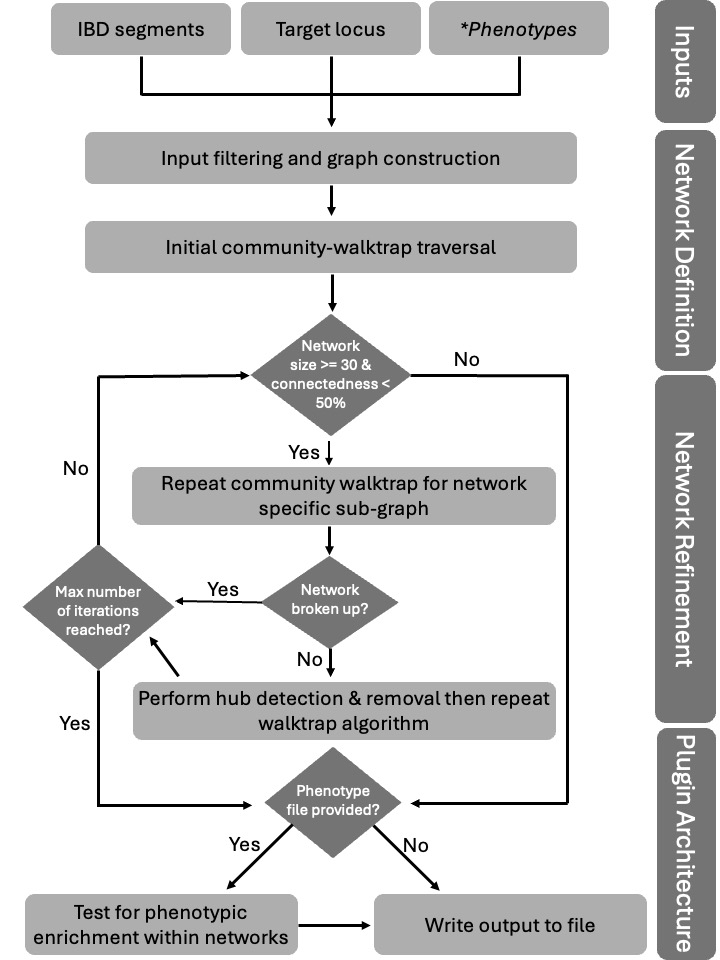


**Influences on runtime and memory behavior:**

The flamegraph (Figure S6B) for DRIVE v3 indicates that a large proportion of runtime comes from the walktrap algorithm (43.98%). This algorithm has an average runtime of O(n^2^log n) and worst-case runtime of O(mn^2^) where n is the number of vertices and m is the number of edges in the graph. In DRIVE, *n* represents the number of unique haplotypes in the network and *m* represents the number of pairwise segments identified in that network. Runtime will scale as both the number of pairwise segments and number of individuals in the entire cohort grows. Larger target region size will likely include more individuals who share pairwise segments overlapping that target sequence causing longer runtimes.

**Hub Detection Algorithm:**

1. Select network to examine. All nodes in networks that meet network size and connectedness criteria (Figure S1) are examined. For illustration purpose we show the testing for a single node of interest (red).

**Figure S2.** Network of 31 individuals with an overall connectedness of 38.2% (178 edges / 465 potential edges). The node highlighted red is the focus of this algorithmic description and functions as a potential hub node connecting 2 subclusters


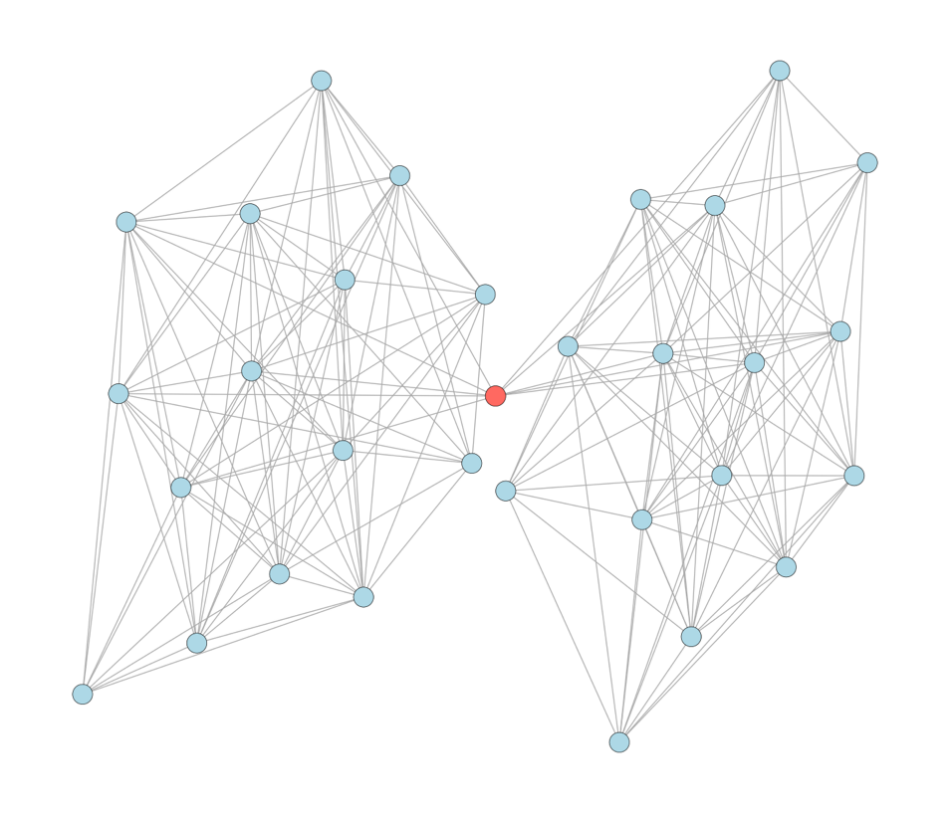


2. Calculate the number of edges connected to the node of interest.


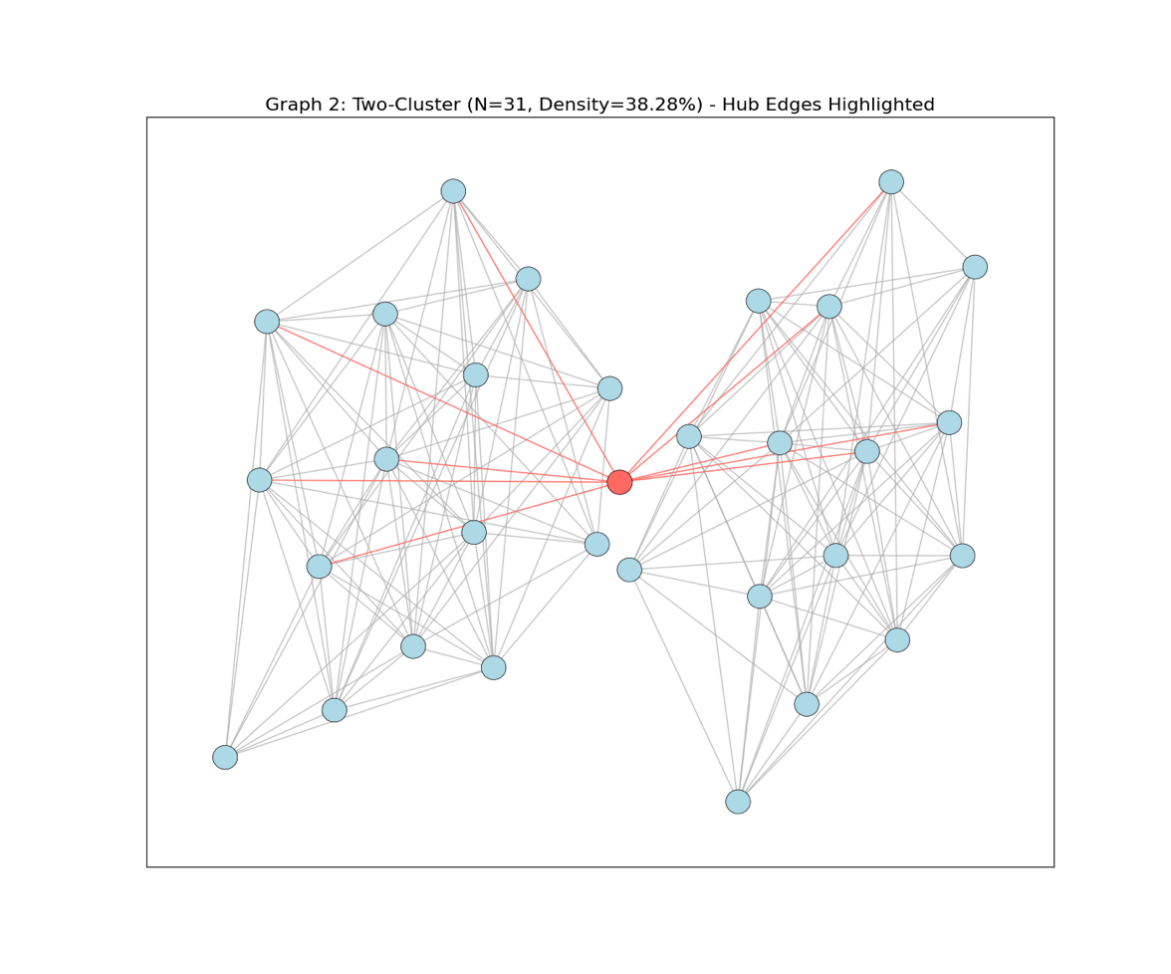


Edge Count = 10

**Figure S3.** To highlight how sparsely the node of interest is connected to the graph, all edges containing that node are highlighted in red

1. Generate penalization term for all nodes in the network by summing the inverse IBD segment length of every edge connected to the node of interest (**Eq 1**).

$penalization score= \sum\frac{1}{pairwise IBD segment length}$ (1)

After the score is calculated, we check to see if the penalization score for the node of interest falls within the top 1% of the distribution for the network (**Figure S4**).

**To illustrate this step, we will generate ‘fake” IBD segment lengths for the network of interest. We will assume that the length of the IBD segments containing the hub node (red) are all ~3 cM and the length of the segments for all other connections are randomly selected values between 3-30cM. These assumptions will over emphasize how much more distantly the hub node is related to other individuals in the network to clearly make a distinction between the hub node and the other nodes.*

**Figure S4.** Distribution of penalization scores (**Eq. 1**) for the network of interest. A larger penalization score indicates the node is more distantly related to others in the network. The score for the hub node is indicated by the red dashed lines.


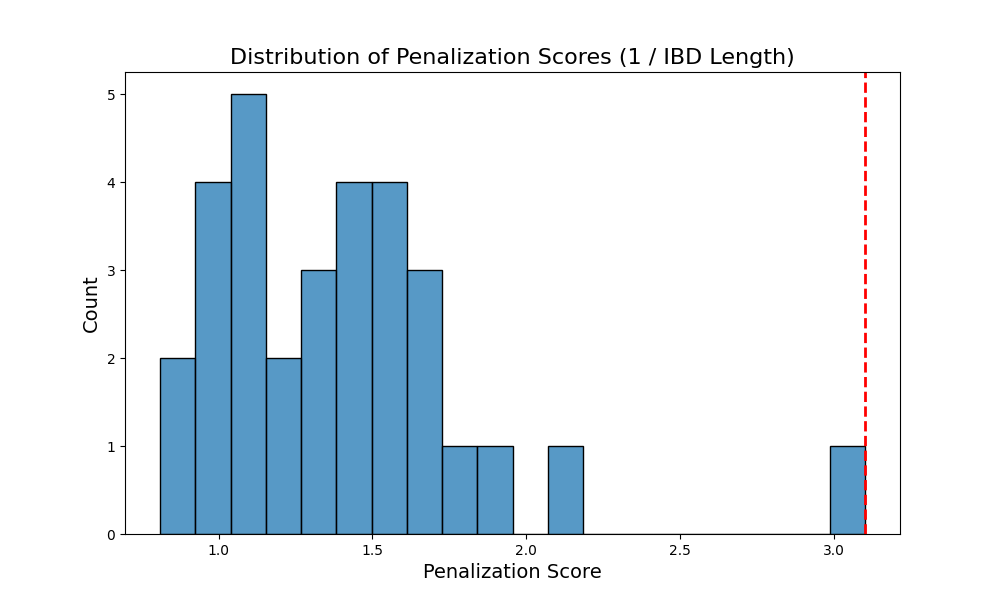


1. If the node of interest meets the following two conditions, then it is classified as a hub.
   1. Connected to >= 20% of the network
   2. the penalization score falls in the tail of the distribution for the network (default ≥ top 1% of scores)
2. Iteratively repeat this process for all nodes in the graph to generate a list of hub nodes.
3. All identified “hub” nodes are removed from the graph.
4. Another random walk is performed to break up the networks (**Figure S5**).

Network 1.2

Network 1.1


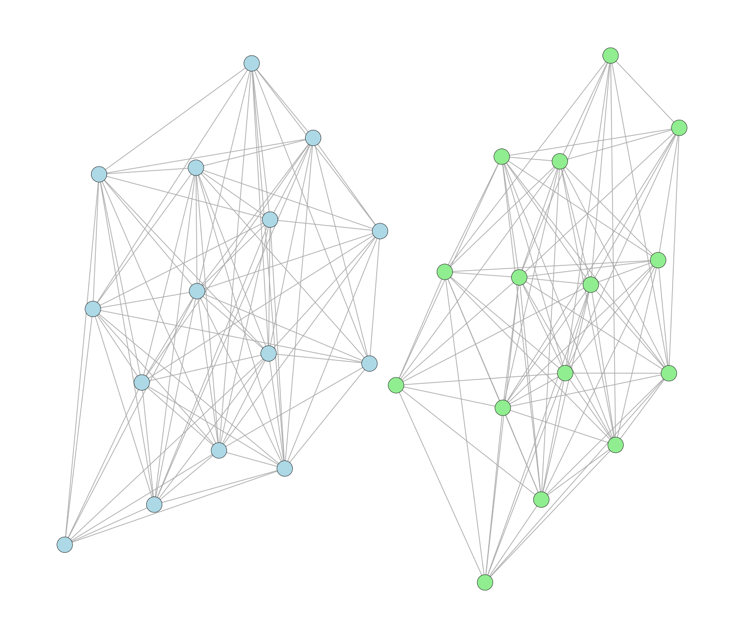


**Figure S5.** After the additional random walk, the formerly one network is now identified as two separate networks. In output files these networks would be listed as derivatives of the original network Ex. If the original network id was 1 then after the hub detection algorithm the new ids are 1.1 and 1.2

**
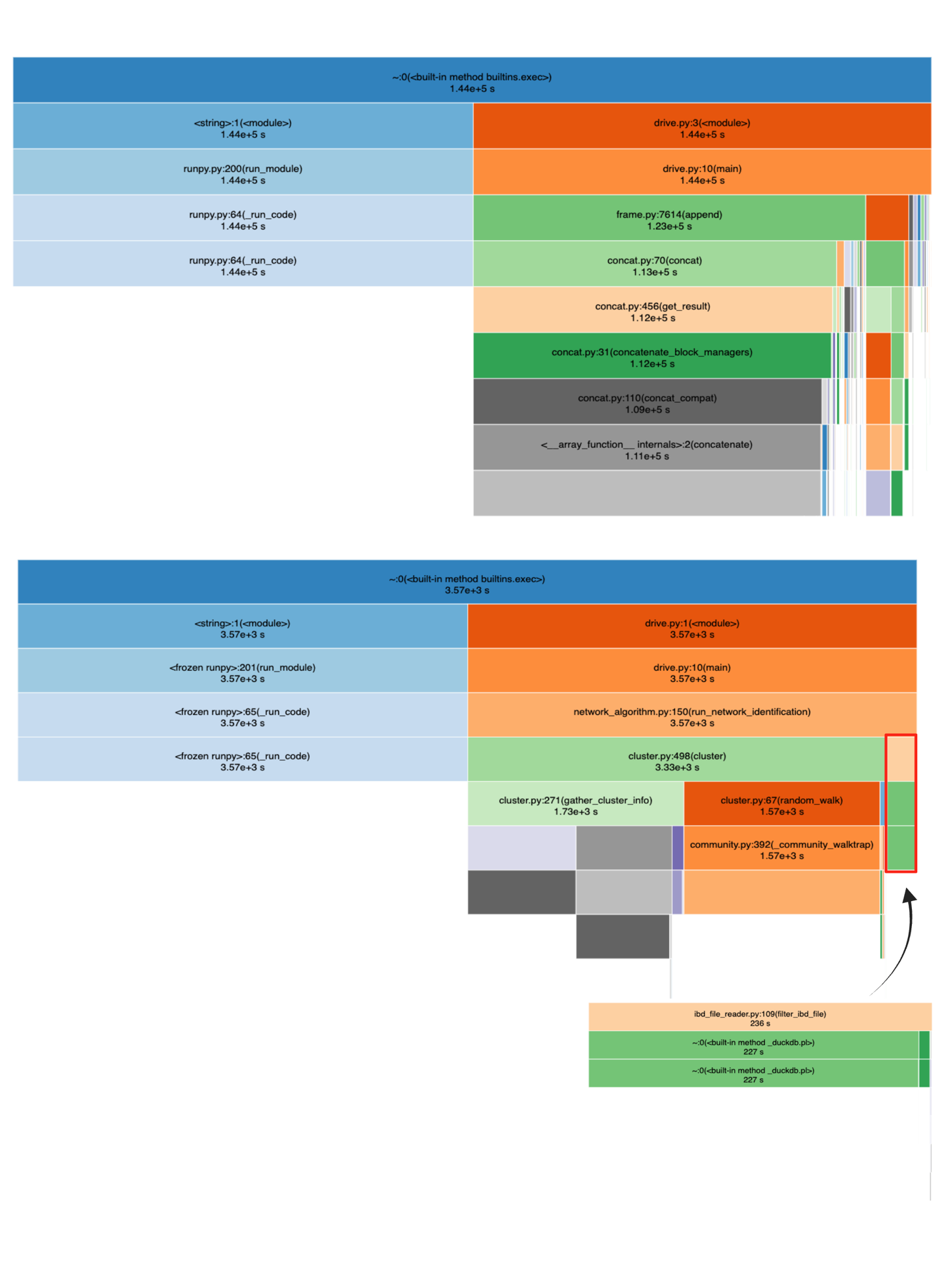
**

B.

**Figure S6.** Flamegraphs showing the runtime call stack and time spent in different functions of both DRIVE version. *A)* DRIVE v1 call stack. *B)* DRIVE v3 call stack. We have highlighted and expanded the IBD parsing and filtering code in the red square.

A.

| **Flag** | **Command Description** | **Default Value** |
| --- | --- | --- |
| --min-cm | Minimum Centimorgan length threshold for the pairwise IBD segments | 3 |
| --step | Number of minimum steps the random walk takes to cluster nodes into a network | 3 |
| --max-recheck | Maximum number of iterations where DRIVE will attempt to re-cluster the data | 5 |
| --max-network-size | Maximum network size threshold to determine if a network should be re-clustered | 30 |
| --min-connected-threshold | Connected threshold used to determine if a network should be re-clustered | 0.5 |
| --min-network-size | Minimum size threshold for DRIVE to identify a network | 3 |
| --hub-threshold | Proportion of top nodes scores that each node is compared to in order to determine if a node is a “hub” and should be removed | 0.01 |
| --segment-distribution-threshold | Threshold for the proportion of total connections that each individual node should have before being classified as a “hub” | 0.2 |

**Table S1**. Descriptions of flags that can be passed to DRIVE to customize the clustering algorithm during runtime.

**Identification of compound heterozygous variant for additional three ΔF508 carriers**

*Haplotype containing chr7:117587778:G:T*

DRIVE identified an additional network of 23 participants, which contained the case carrying variant chr7:117587778:G:T. All 23 participants in the network were sequenced as carriers for this variant. This network contained an additional 2 CF cases, 1 of which was also sequenced also as a carrier for the ΔF508 variant while the other individual was not sequenced for any other pathogenic CF variant.

*Haplotype containing chr7:117587811:C:T*

DRIVE identified an additional network of 11 participants, which contained the case carrying variant chr7:117587811:C:T. All participants in the network were sequenced as carriers for this variant. This network contained an additional 2 CF cases, both of which were sequenced as carriers for the ΔF508 variant.

*Haplotype containing chr7:117590440:G:A*

DRIVE identified an additional network of 69 individuals containing CF case carrying the variant chr7:117590440:G:A. All individuals within this network were sequenced as carriers for the variant chr7:117590440:G:A. This network contained 6 additional CF cases, of which 5 participants carried an additional pathogenic variant while 1 individual was only sequenced for the chr7:117590440:G:A variant.
